# Effects of BYL-719 (alpelisib) on human breast cancer stem cells to overcome drug resistance in human breast cancer

**DOI:** 10.3389/fphar.2024.1443422

**Published:** 2024-10-14

**Authors:** Leinan Yu, Chuanbing Zang, Yuanchun Ye, Hongyu Liu, Jan Eucker

**Affiliations:** ^1^ Department of Hematology, Oncology and Cancer Immunology, Campus Benjamin Franklin Charité ‐ Universitätsmedizin Berlin, Corporate Member of Freie Universität Berlin and Humboldt‐Universität zu Berlin, Berlin, Germany; ^2^ School of Science, Shenzhen Campus of Sun Yat-Sen University, Shenzhen, Guangdong, China; ^3^ Department of Oncology, Rheumatology and Gastroenterology, Vivantes Klinikum Spandau, Berlin, Germany

**Keywords:** breast cancer, breast cancer stem cell, BYL-719 (alpelisib), PI3K/Akt signaling pathway, stemness, resistance

## Abstract

**Introduction:**

Breast cancer continues to be a major health concern and is currently the most commonly diagnosed cancer worldwide. Relapse, metastasis, and therapy resistance are major clinical issues that doctors need to address. We believe BYL-719, which is PI3 kinase p110а inhibitor, could also inhibit the breast cancer stem cell phenotype and epithelial-to-mesenchymal transition (EMT). In addition to the PI3K/AKT signaling pathway, BYL-719 can also inhibit essential cancer-related signaling pathways, all of which would ultimately act on the microenvironment of cancer stem cells, which is quite complicated and regulates the characteristics of tumors. These include the stemness and resistance of malignant tumors, plasticity of cancer stem cells, and anti-apoptotic features.

**Materials and methods:**

A three-dimensional (3D) mammosphere culture method was used *in vitro* to culture and collect breast cancer stem cells (BCSCs). MTT, clonogenic, and cell apoptosis assays were used to detect cell viability, self-renewal, and differentiation abilities. A sphere formation assay under 3D conditions was used to detect the mammophore inhibition rate of BYL-719. The subpopulation of CD44^+^CD24^−^ was detected using flow cytometry analysis while EMT biomarkers and essential signaling pathways were detected using western blotting. All the data were analyzed using GraphPad Prism 9 software.

**Results:**

BCSC-like cells were obtained by using the 3D cell culture method *in vitro*. We confirmed that BYL-719 could inhibit BCSC-like cell proliferation in 3D cultures and that the stemness characteristics of BCSC-like cells were inhibited. The PI3K/AKT/mTOR signaling pathway could be inhibited by BYL-719, and the Notch, JAK-STAT and MAPK/ERK signaling pathways which have crosstalk in the tumor microenvironment (TME) are also inhibited. By comparing eribulin-resistant breast cancer cell lines, we confirmed that BYL-719 could effectively overcome drug resistance.

**Summary/conclusion:**

The 3D cell culture is a novel and highly effective method for enriching BCSCs *in vitro*. Furthermore, the stemness and EMT of BCSCs were inhibited by BYL-719 by acting on various signaling pathways. Finally, we believe that drug resistance can be overcome by targeting the BCSCs. Conjugation of BYL-719 with other anti-neoplastic agents may be a promising treatment for this in clinic.

## 1 Introduction

Breast cancer continues to be a serious global public health issue, with an unprecedented impact on human lifespan and health (Sung et al., 2021; Wilkinson and Gathani, 2022). Over the last few decades, thousands of scientists have focused on the mechanisms and comprehensive therapies of breast cancer, and their research has made substantial progress in our understanding of the disease (Wang Z. et al., 2024). They have revealed that the main factors contributing to breast cancer include aging, family history, reproductive factors, estrogen, progesterone, and lifestyles (Sun et al., 2017). The conventional treatments for breast cancer comprise surgery, radiotherapy, chemotherapy, endocrine therapy, neoadjuvant therapy, and adjuvant therapy (Wang and Wu, 2023; Zhang et al., 2023). Although different treatment methods are available, metastasis, relapse, and resistance are the usual problems that patients and doctors face after several years of treatment. These problems lead to a lowered 5-year survival rate and reduced quality of life later on (Taskindoust et al., 2021). As research continues to evolve, scientists have found that one of the most important reasons for these problems is the presence of breast cancer stem cells (BCSCs) (De Angelis et al., 2019). They have elucidated the concept and function of BCSCs, emphasizing their existence in humans for long periods and their high plasticity along with self-renewal properties (Zhang et al., 2020). In 2006, the American Association for Cancer Research defined a CSC as a cell within a tumor that possesses the capacity to self-renew and cause heterogeneous lineages of cancer cells that comprise a tumor (Najafi et al., 2019). It is well known that special proteins that determine the key phenotype can be used as markers for specific cells (Luo et al., 2024). Currently, BCSCs are usually identified by expression of specific phenotypes; CD44^+^/CD24^−^/low and/or CD133^+^ are most frequently used (Li et al., 2017), and it is identified as a small subpopulation of heterogeneous breast cancer cells with strong self-renewal and proliferation properties (Zhang et al., 2020). The major putative mechanisms underlying the properties of BCSCs include the tumor microenvironment (TME), stem cell-related signaling pathways, enhancement of epithelial-to-mesenchymal transition (EMT) cellular programming, DNA damage and repair pathways, as well as miRNA and epigenetic alterations (Nilendu et al., 2018; Zhang et al., 2022). Although highly proliferative, BCSCs predominantly remain in a quiescent state or cycle slowly, shielding them from chemotherapy and radiation damage. Collectively, these factors contribute to the survival of BCSCs during treatment and their ability to re-establish tumor masses post-therapy.

Commercially available inhibitors target both membrane proteins and BCSC-related signaling pathways. BYL-719 (alpelisib) is an inhibitor of phosphatidylinositol 3-kinase (PI3K) that has substantial anticancer action (Markham, 2019). It functions by specifically inhibiting class I PI3K p110α, the catalytic subunit of PI3K, a lipid kinase involved in numerous biological processes, including proliferation, survival, differentiation, and metabolism. Patients treated with alpelisib have shown better tolerance and longer progression-free survival (PFS) (Markham, 2019). Alpelisib also possesses favorable pharmacokinetic properties, characterized by rapid and significant absorption (Marbury et al., 2023). Currently, there are no data on the effects of PI3K inhibitors on BCSC-like cells (Chang et al., 2021). In our research, alpelisib was established as a highly effective PI3Kа inhibitor which could also affect the BCSCs and interrupt the crosstalk between signaling pathways including Notch, JAK/STAT and MAPK/ERK signaling pathways and studies have already showed there exits cross-talks in TME of BCSC, which is a complicated microenvironment includes intrinsic and extrinsic factors. This suggests that between these important signaling pathways and all these molecules have inter-linkages and interactions. As previously described, these intricate signal transduction pathways are not linear. The PI3K/AKT/mTOR signaling pathway is responsible for the promotion of cell proliferation, survival, and cell cycle progression (Glaviano et al., 2023). Notch inhibits the proliferation and differentiation of CSCs, thus maintains the CSC phenotype and contributes to the transformation process (Martínez-Pérez et al., 2024). The JAK-STAT pathway is always considered to have a role which could regulate the survival and proliferation of BCSCs.It is also believed to be associated with metastasis and drug resistance. The signal transducer and activator of transcription (STAT) protein family plays a major role in cancer (Liongue et al., 2024). Mitogen-activated protein kinase (MAPK) cascades is important to the cellular processes, including differentiation, apoptosis, proliferation, and responses to stress. It is one of the most critical cancer related signaling pathways (Guo et al., 2020). Furthermore, it is now confirmed that the activation of extracellular signal-regulated kinase (ERK) leads to the formation of spheres and the CSC-like properties (Choi et al., 2018). Additionally, it can block the TME to disrupt stem cell characteristics, such as self-renewal therapeutic resistance, tumor recurrence, and metastasis (Fruman et al., 2017). Now it is confirmed that Notch signaling is related with self-renewal ability, activating of PI3K signaling leads to enhanced antiapoptotic ability, JAK-STAT signaling leads to tumor progression and drug resistance (Butti et al., 2019). Together with tumor microenvironment-sustaining effects (exosomes or chemokines), these factors could contribute to a therapy-resistant phenotype of BCSC, highlighting the importance of precision treatment approaches in managing complex cancers (Yang et al., 2024).

It has already been showen in much cancer research that the three-dimensional (3D) cell culture models could provide an overview of cell-to-cell communication and interactions (Habanjar et al., 2021). Moreover, 3D cell culture models can reproduce important aspects of tumor structure and microenvironment, and also help to reduce the use of laboratory animals in drug trials (Barbosa et al., 2021). Using the 3D cell culture method, we confirmed that BYL-719 could effectively overcome drug resistance by inhibiting BCSCs, which may be a prominent clinical tool in the future. Our experimental methods and research ideas were innovative. However, there is still a need for in-depth studies on BYL-719 and the mechanisms of overcoming breast cancer resistance, as there is not much similar research currently available.

## 2 Materials and methods

### 2.1 Cell culture

Human breast cancer cell lines, MCF-7 and T47D, were purchased from the American Type Culture Collection (Rockville, MD, United States). The cells were grown in RPMI 1640 (Gibco) containing 10% fetal bovine serum (Gibco), with penicillin (100 U/mL), and streptomycin B (100 mg/mL). All cells were cultured in a 5% CO_2_ incubator at 37°C with 5% relative humidity (Zhao et al., 2024).

### 2.2 Three-dimensional (3D) mammosphere culture method

Cells (1,000/cm^2^ cells per well) were added to a low attachment six-well plate in serum free DMED/F12 medium (Corning, United States) supplemented with 2 mM L-glutamine, 100 U/mL penicillin/streptomycin, 20 ng/mL EGF (90201, BPS Bioscience), 10 ng/mL FGF (3718-FB-100, Biotechne), 2.5% Matrigel (Corning, United States) and 1× B27 supplement (17504044, Gibco). The plates were incubated for 5–7 days until the mammospheres (>40 µm) were formed. The mammospheres from each well were then collected. After slow centrifugation, the spheres were trypsinized for 2–3 min to separate them into single-cell suspensions. After at least five repetitions, we collected the enriched BCSC-like cells for the experiments by the 3D culture method (Lee et al., 2023).

### 2.3 Cells viability via 3-(4,5-dimethylthiazol-2-yl)-2,5-diphenyltetrazolium bromide (MTT) assay

To determine the dose response to BYL-719 (S2814, Selleck, United States), cells were seeded in a 96-well plate in six replicates at the density of 2 × 10^3^/well and incubated overnight, then treated with serial dilutions of BYL-719 at 37°C for 96 h. After 96 h treatment, cells were incubated with 10 μL yellow MTT solution (Cell Proliferation Kit I (MTT), Roche) for 2–3 h in the incubator (Zhao et al., 2020). The 100 μL solubilization solution was then added and the plate was placed overnight in the incubator in a humidified atmosphere. Absorbance of the formazan product was measured at 490 nm using a microplate reader.

### 2.4 Clongenic assay (2D)

One thousand cells per well in were added to a 12-well plate with 1 mL DMED/F12 medium supplemented with 2 mM L-glutamine, 100 U/mL penicillin/streptomycin, 20 ng/mL EGF, 10 ng/mL FGF, and 1× B27 supplement containing different concentrations of BYL-719. After 7 days, the medium was replaced with 1 mL fresh medium with appropriate concentrations of BYL-719 and incubated for another 7 days. After 2 weeks of incubation, cell colonies (Brix et al., 2021) were visualized using Quick staining (Merck, Darmstadt, Germany) and photographed.

### 2.5 Mammospheres forming assay

Five hundred cells per well were added to a 24-well low attachment plate in 500 µL mammoshpere media are added to each well and incubated with different concentrations of BYL-719 for 5 days. The mammospheres (diameter >40 µm) were counted and mammosphere forming efficiency (Lombardo et al., 2015) were calculated as percentage of cells seeded and recorded.

### 2.6 Sphere-formation assay (3D condition)

Two thousand cells/well in with 100 µL mammosphere media with appropriate concentrations of BYL-719 were added to a 96-well U bottom low attachment plate. The cells were cultured for 10 days. After incubation, the diameters of the spheres in each well were measured and compared with those in control wells.

### 2.7 Flow cytometry analysis to detect biomarkers of BCSCs

The reversed cells were digested by Accutase. Cells were washed, blocked with FC block (1:50), centrifuged and resuspended in 100 μL fluorescence-activated cell sorting (FACS) buffer (PBS containing 0.5% BSA and 0.1% sodium azide), containing fluorochrome-conjugated monoclonal antibodies against human CD44 (FITC, 555478, BD Biosciences) at 1:80 dilution and CD24 (PE, 555428, BD Biosciences) at 1:20 dilution. The cells were then washed again with cold PBS, suspended, filtered through 40-µm nylon mesh before analysis and measured with a CytoFlex flow cytometer (Beckman, United States).

### 2.8 Cell apoptosis via annexin V/PI assay

Cells were seeded overnight and treated with 1 μM concentration of BYL-719. After 24 h drug treatment, cells were detached by 0.25% trypsin, washed and resuspend in 1× binding Buffer at a concentration of 1 × 10^6^ cells/mL. The solution was then transferred to a 5 mL FACS tube. 1 μL of FITC (Annexin V PE Annexin V Apoptosis Detection Kit I) was added, and the cells were gently vortexed and incubated at RT (25°C) for 15 min in the dark. The 1× Binding Buffer (400 μL) and 2 μL PI were added to each tube. Apoptosis was analyzed using flow cytometry and the FlowJo software (V10).

### 2.9 Western blotting

The extracted proteins were separated using 10% SDS-PAGE gels. Blots were incubated at 4°C overnight with the primary antibodies against NANOG (#4903, 1:2,000, Cell Signaling Technology), OCT3/4 (#365509, 1:1,000, Santa Cruz), Sox2 (#3579, 1:1,000, Cell Signaling Technology), EMT (#9782, 1:500, Cell Signaling Technology), p-4ebp1 (#2855, 1:500, Cell Signaling Technology), p-P70S6k (#9205, 1:500, Cell Signaling Technology), p-AKT (#9271, 1:500, Cell Signaling Technology), and pARP (#9542, 1:500, Cell Signaling Technology), the Notch Activated Targets Antibody Sample Kit (#68309, 1:1,000, Cell Signaling Technology). Secondary antibodies (Santa Cruz) were then used and detected using ECL Prime Western Blotting Detection Reagent (GE Healthcare). Images were obtained using the ImageJ software (Wang J. et al., 2024).

### 2.10 Statistics

Statistical analyses were performed using the GraphPad Prism 9 software (GraphPad Software, La Jolla, United States). Shapiro–Wilk and Kolmogorov–Smirnov tests were used for normal distribution analysis. Unpaired t-tests (with Welch’s correction in data without equal variances) were used for two independent data sets. One-way ANOVA (with Welch’s correction in data without equal variances) and Tukey’s multiple comparisons test were used for more than two independent samples. Half-maximal IC50 values were calculated using non-linear regression analysis. Statistical significance was set at *P* < 0.05. significant. The following symbols were used: ns, non-significant; *P < 0.05, **p < 0.01, ***p < 0.001, and ****p < 0.0001. Error bars represent the standard error. The 95% confidence interval (CI) were calculated.

## 3 Results

### 3.1 BYL-719 inhibits cell viability of breast cancer cells and mammospheres

**FIGURE 1:**
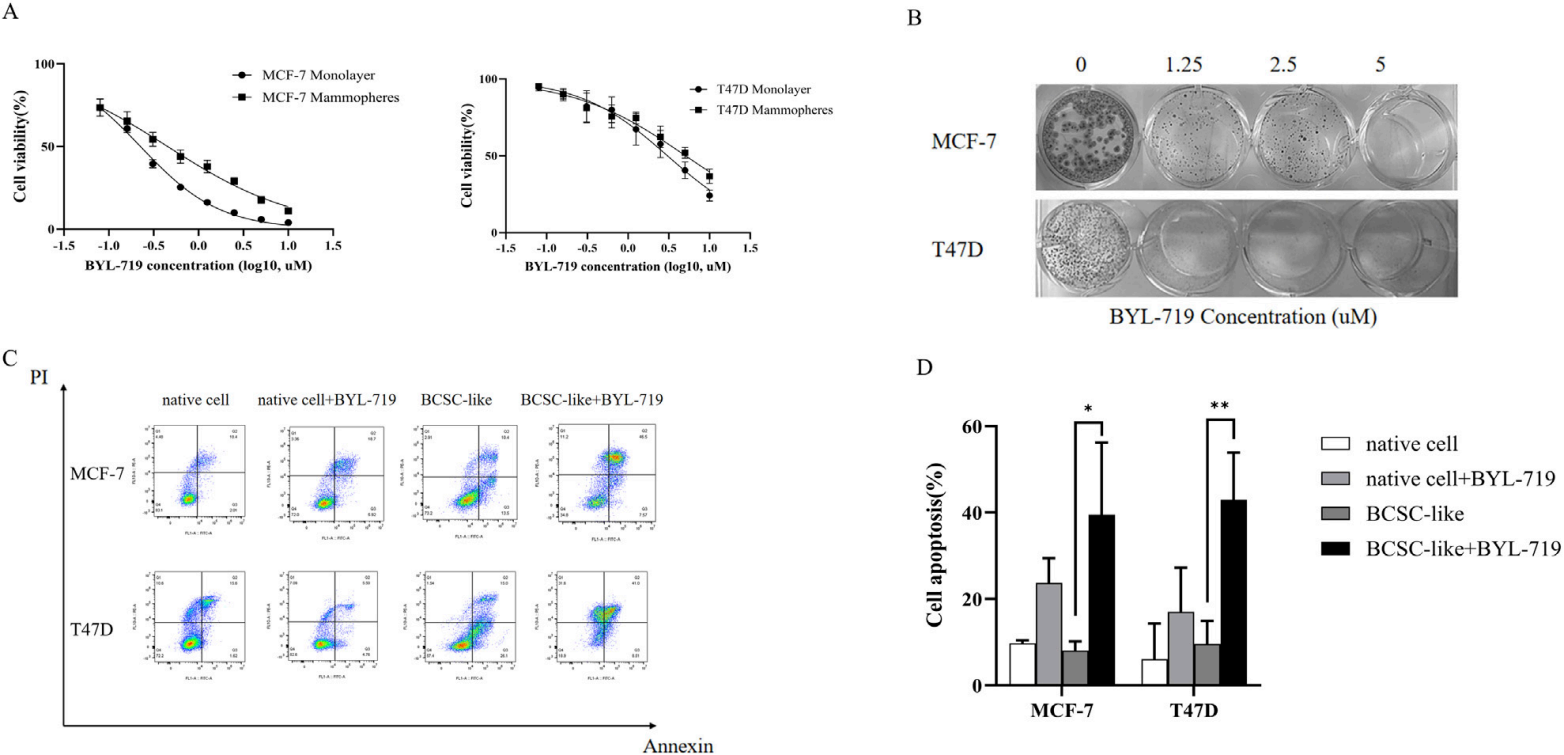
BYL-719 inhibits cell viability of breast cancer cells. **(A)** Breast cancer cells propagated as monolayers (differentiated cultures, solid line) or mammospheres (enriched in BCSCs, dotted line) were treated with the indicated concentrations of BYL-719 for 96 h. The % viability of cells at each doxycycline dose at the end of treatment was measured using a Cell Proliferation Kit I (MTT). The IC50 (MCF7 and T47D was 0.225 μM and 3.055 μM, respectively). **(B)** BYL-719 inhibited the clonal growth of breast cancer cell lines in clonogenic assays (2D) in both MCF-7 and T47D cell lines. **(C, D)** BYL-719 treatment induced early (Annexin-PI) apoptotic cell death in both breast cancer lines. Ns for non-significant,* for *P* < 0.05, ** for *P* < 0.01, *** for *P* < 0.001 and **** for *P* < 0.0001. Errors bars represent standard errors.

MCF-7 and T47D human breast cancer cells were treated with increasing concentrations of BYL-719 for 96 h, and the effect of BYL-719 on cell viability was measured. BYL-719 inhibited viability of breast cancer cells in a dose-dependent manner with IC50 values (concentration of drug that inhibits 50% of cell viability relative to untreated cells) for MCF-7 and T47D of 0.225 μM and 3.055 μM, respectively (Figure 1A). In addition, treatment with BYL-719 significantly inhibited proliferation of BCSC-enriched mammosphere cultures 96 h after a single treatment (Figure 1A). However, in agreement with other studies demonstrating that BCSCs are in general more resistant to anticancer drugs, the IC50 values for BCSC-enriched mammosphere cultures increased approximately two-fold for both lines (0.453 μM and 5.105 μM for MCF-7 and T47D, respectively) (Figure 1A). We further confirmed the toxic effects of BYL-719 via clonogenic assays and assessed its impact on the cellular self-renewal capacity. The number of colonies formed with different concentrations of BYL-719 was significantly reduced compared to that of the controls in MCF-7 and T47D cells (Figure 1B). Colonies were fixed and visualized using quick stain. Finally, by using Annexin-PI straining and FACS analysis to detect the apoptosis, we showed that BYL-719 of the concentration of 1 µM induced apoptosis especially in BCSC-like cell populations (Figure 1C). Apoptotic cells in the BYL-719 treated group of BCSC-like cell populations were significantly increased compared to those in the BCSC-like cell groups in both cell lines (MCF-7: *p* = 0.00107, 95% CI, 8.246 to 54.77; T47d: *p* = 0.0079, 95% CI, 9.976–56.72) (Figure 1C). These results suggest that BYL-719 can activate and promote cell death signaling pathways, including autophagy, apoptosis, ferroptosis, and necroptosis, through crosstalk among the BCSC signaling pathways.

### 3.2 BYL-719 inhibits stem cell marker expression and self-renewal in breast cancer stem cells

**FIGURE 2:**
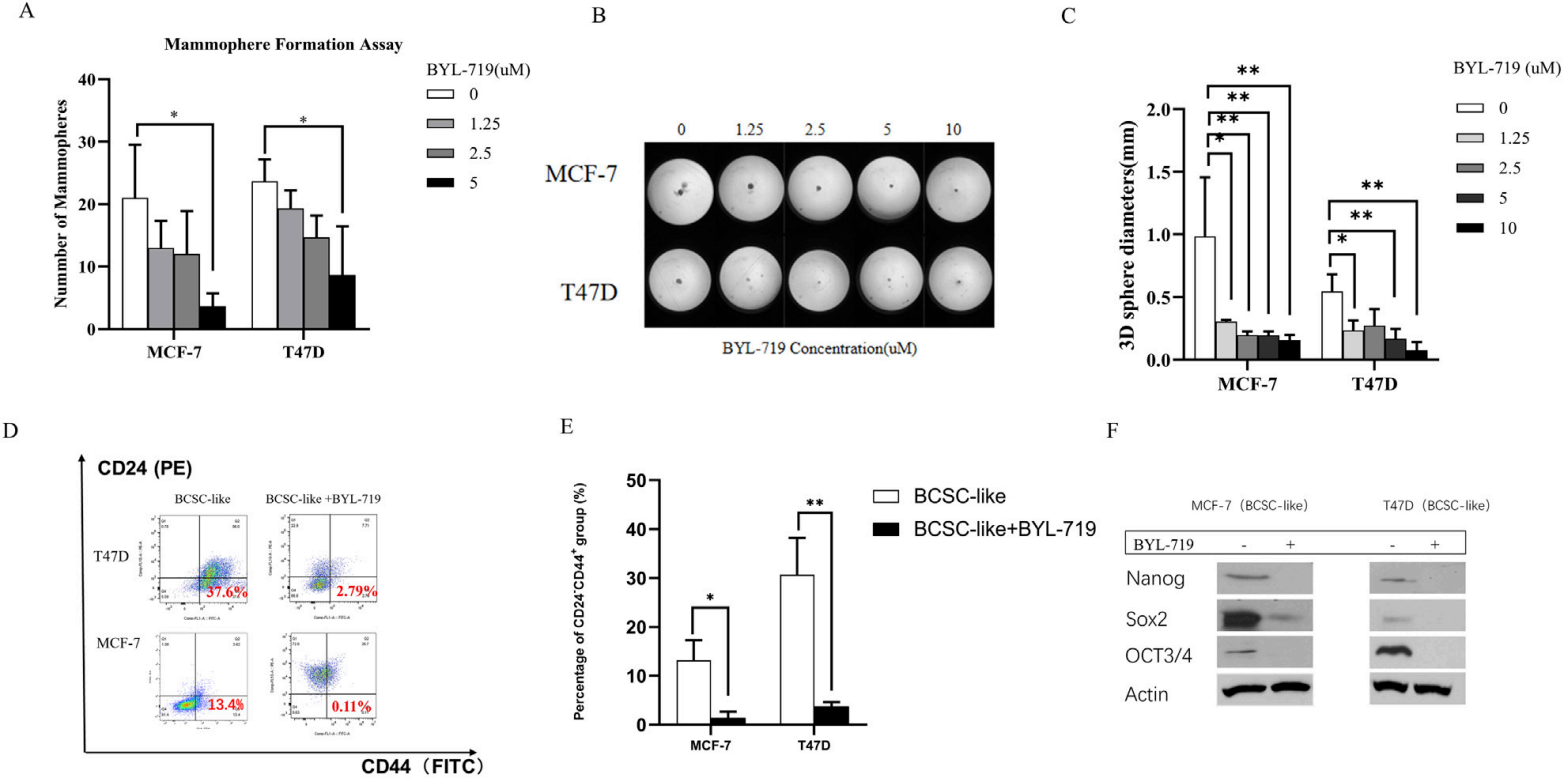
BYL-719 decreased the EMT phenotype in both breast cancer cell lines. **(A)** MFEs of BCSC-like cells were decreased by BYL-719 dose-dependently. **(B, C)** Sphere-forming abilities were inhibited by BYL-719 in MCF-7 and T47D cell lines. **(D, E)** BYL-719 reduced the percentages of CD24^−^CD44^+^ in BCSC enriched cell. **(F)** Western blot analysis for EMI-related proteins. Treated groups were treated with BYL-719 1 μM for 24 h. Ns for non-significant, * for *P* < 0.05, ** for *P* < 0.01, *** for *P* < 0.001 and **** for *P* < 0.0001. Errors bars represent standard errors.

The self-renewal capacity of BCSCs in both breast cancer lines was measured using a mammosphere-forming efficiency (MFE) assay. The MFE assay with BCSC-enriched cell populations showed a strong dose-dependent reduction in MFEs by BYL-719 in both cell lines. By the concentration of 5 μM, MFE decreased in both cell lines (MFE for MCF-7, *p* = 0.047, 95% CI, 1.627–33.04; MFE for T47D, *p* = 0.0262, 95% CI, 2.370–27.63) (Figure 2A). Next, we performed a 3D sphere-forming assay using different concentrations of BYL-719 to determine its effects of BYL-719 in 3D condition. After incubation, the spheres formed in each well were photographed, and their diameters were calculated using GraphPad Prism 9 software (Figure 2B). In both cell lines, BYL-719 significantly reduced sphere diameter, indicating that stemness and resistance could be inhibited by BYL-719 (Figure 2C). We also investigated the effect of BYL-719 on the BCSC population using a combination of surface markers for BCSCs. The CD44^+^CD24^−^ cell population has been shown to identify a subpopulation of cells in breast cancer enriched for BCSCs. This is illustrated in Figure 2D. Before the FACS analysis, each group was treated with BYL-719 at a concentration of 1 μM for 24 h. After treatment with BYL-719, the CD44^+^CD24^−^ cell populations present in BCSCs significantly decreased compared to those in untreated cells (MCF-7, *p* = 0.0088, 95% CI, 4.941–18.64; T47D, *p* = 0.0237, 95% CI, 8.631–45.32) (Figure 2E). Inhibition of stem cell factors at the gene level was accompanied by lower protein levels after treatment with 1 μM BYL-719 for 24 h compared to that in untreated controls (Figure 2F). Nanog, Sox2, and OCT3/4 levels were significantly decreased in both MCF-7 and T47D cell lines; for the MCF-7 cell line, Nanog (*p* <0.001; 95% CI, 0.9908–1.045); Sox2 (*p* = 0.0069, 95% CI, 0.5014–1.062); OCT3/4 (*p* = 0.0014, 95% CI, 0.5370–0.7396), and for T47D cell line, Nanog (*p* = 0.0007, 95% CI, 0.6007–0.7528); Sox2 (*p* = 0.0047, 95% CI, 0.5294–0.9732); and OCT3/4 (*p* = 0.0026, 95% CI, 0.7925–1.239).

### 3.3 BYL-719 inhibits various important signaling pathways

**FIGURE 3:**
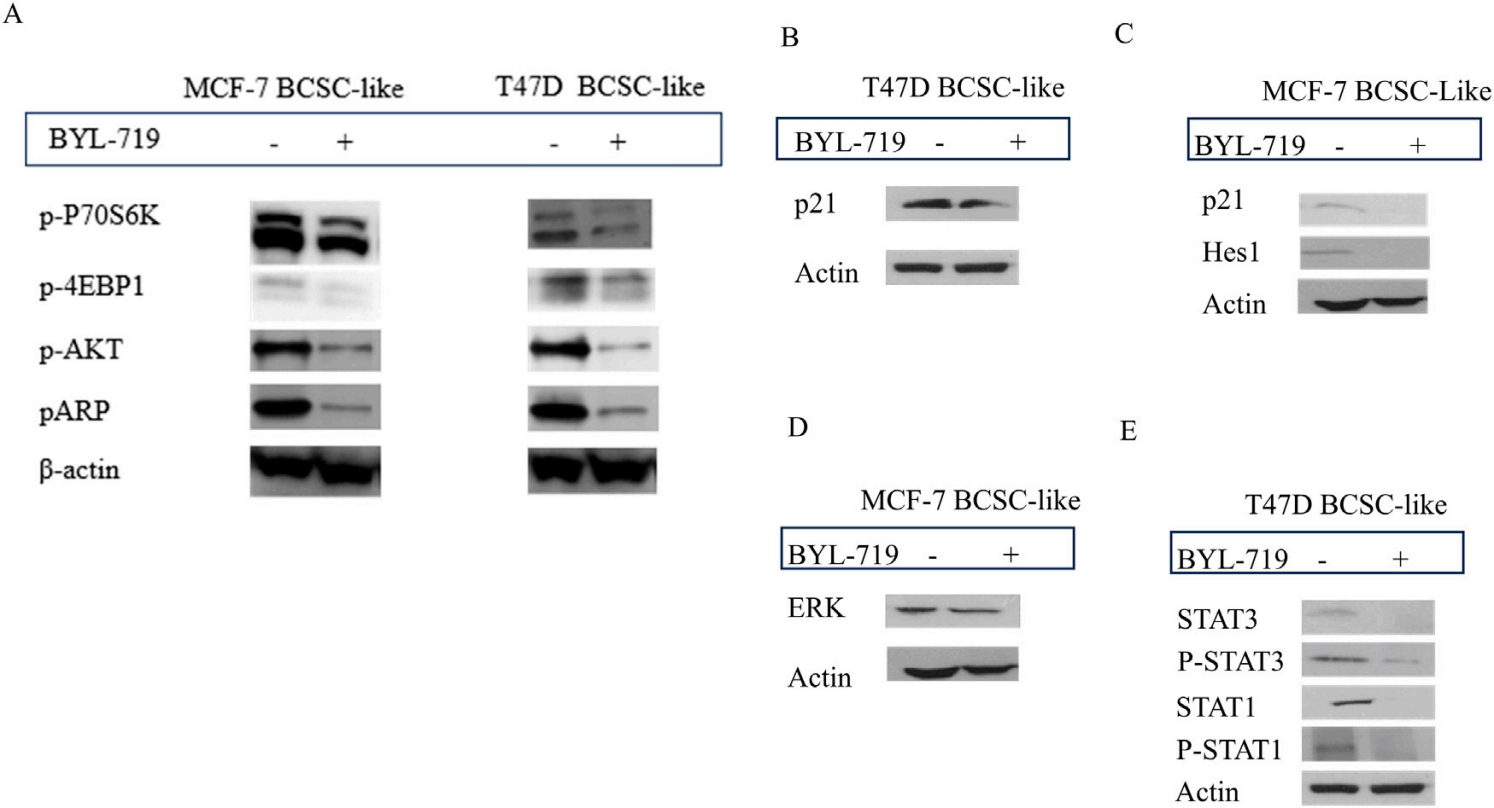
The inhibition of BYL-719 in PI3K/AKTATOR: Notch; MAPK/ERK; JAK/STAT signaling pathways. All the treated groups were treated with BYL-719 of the concentration of 1 μM for 24 h. **(A)** BYL-719 inhibited PI3K/AKT/mTOR signaling pathway in MCF-7 BCSC-like treated group and T47D BCSC-like treated group **(B, C)** Notch signaling pathway and p21 protein were inhibited by BYL-719 in MCF-7 BCSC-like treated group and T47D BCSC-like treated group. **(D)** ERK was decreased in MCF-7 BCSC-like treated group. **(E)** JAK/STAT signaling pathway was inhibited by BYL-719 in T47D BCSC-like treated group. Image **(B)** and Image **(E)** are from the same cells in a same experiment, with the same loading control. Image **(C)** and Image **(D)** are from the same cells in a same experiment, with the same loading control. We seperate these images in order to explain the changes in different signaling pathways more clearly.

Western blot analysis demonstrated that the PI3K/AKT/mTOR inhibitor alpelisib (BYL-719) was highly effective. By comparing BCSC-like group and BYL-719 treated group (1 μM, 24 h), the downstream proteins changed significantly (Figure 3A). We detected a significant rise in BCSC-like group than native cell, in MCF-7 cell line, p-P70S6K (*p* = 0.0059, 95% CI, 0.5835–1.167); p-4EBP1 (*p* = 0.0296; 95% CI, 0.1505–1.093); p-AKT (*p* = 0.0457; 95% CI, 0.0316–1.304); pARP (*p* = 0.0437; 95% CI, 0.04852–1.334). And in T47D cell line, p-P70S6K (*p* = 0.0498; 95% CI, 0.0020–2.362); p-4EBP1 (*p* = 0.0447; 95% CI, 0.05451–1.797); p-AKT (*p* = 0.0371; 95% CI, 0.1561–1.965); and pARP (*p* = 0.0463; 95% CI, 0.0556–2.654). After treatment with BYL-719, the levels of all the downstream protein markers decreased. The decrease in p-P70S6K; p-4EBP1and p-AKT enables us to demonstrate that the PI3K/AKT/mTOR signaling pathway is active in BCSC and is susceptible to efficient inhibition by BYL-719. pARP regulates several activities that are crucial to the functioning of cells, including transcription, apoptosis, and the response to DNA damage.

Other signaling pathways that crosstalk with the PI3K/AKT/mTOR signaling pathway in the TME of BCSCs and act as PI3K inhibitors include the MAPK/ERK, Notch, and STAT signaling pathways. BYL-719 can affect these signaling pathways as well as some intrinsic and extrinsic pathways. The levels of some important proteins in these signaling pathways significantly changed (*P* < 0.05).

In the MAPK/ERK pathway, we detected the ERK protein which has been proved to be associated with the sphere-formation and maintainess of CSC-like characteristics. More importantly, ERK inhibitors are able to overcome the acquired drug resistance induced by upstream kinases inhibitors. In addition, ERK inhibition is the most effective target in MAPK/ERK signaling pathway (Liu et al., 2018). We detected a significant decrease of ERK protein treated with 1 μM BYL-719 for 24 h (*p* = 0.0256; 95% CI, 0.5083–2.904) (Figure 3D). The ERK/MAPK signaling pathway was thought to be inhibited by BYL-719, a widely known PI3K inhibitor. Notably, altered proteins can be found in a variety of cell types. Hes1 have an important function in the maintenance of cancer stem cells self-renewal, cancer metastasis, and epithelial-mesenchymal transition (EMT) process induction, as well as chemotherapy resistance (Liu et al., 2015). In BC, CDKN1A/p21 is induced by the Akt pathway, particularly in HER-2/neu-overexpressing cells, results in cytoplasmic localization in breast cancer cell lines. This is particularly noteworthy. This event is essential for the survival of cancer cells and their resistance to apoptosis. Moreover, the recent research indicate the p21 protein may lead to the chemoresistance. After treatment with the same dose of BYL-719, p21 decreased in both cell lines, and we detected a decrease in Hes1 expression in MCF-7 cells (Figures 3B, C). Although the targeted molecules were different, the trends were the same in both groups, suggesting that the Notch signaling pathway was activated in BCSC-like cells and could be effectively inhibited by the PI3K inhibitor BYL-719. Quantification of p21 (*p* = 0.0340; 95% CI, 0.03491–0.3408) and HES1 (*p* = 0.0418; 95% CI, 0.01394–0.2904) levels significantly reduced after treatment with BYL-719.

In the T47D cell line, p21 was also decreased in the BYL-719 treated group (1 μM, 24 h) (*p* = 0.0237; 95% CI, 0.1697–1.378) and the STAT signaling pathway, which were associated with breast cancer and the stem cells (Figure 3E). STAT3 (*p* = 0.0350; 95% CI, 0.06291–0.6618), P-STAT3 (*p* = 0.0375; 95% CI, 0.04564–0.5930), STAT1 (*p* = 0.0466; 95% CI, 0.03697–1.955), and P-STAT1 (*p* = 0.0481; 95% CI, 0.02025–1.905) were all significantly decreased, corroborating our hypothesis that BYL-719 effectively suppresses the JAK/STAT signaling pathway activity.

### 3.4 Roles of BCSC-like cells in eribulin resistance and effects of BYL-719 in overcoming eribulin resistance in breast cancer cells

**FIGURE 4:**
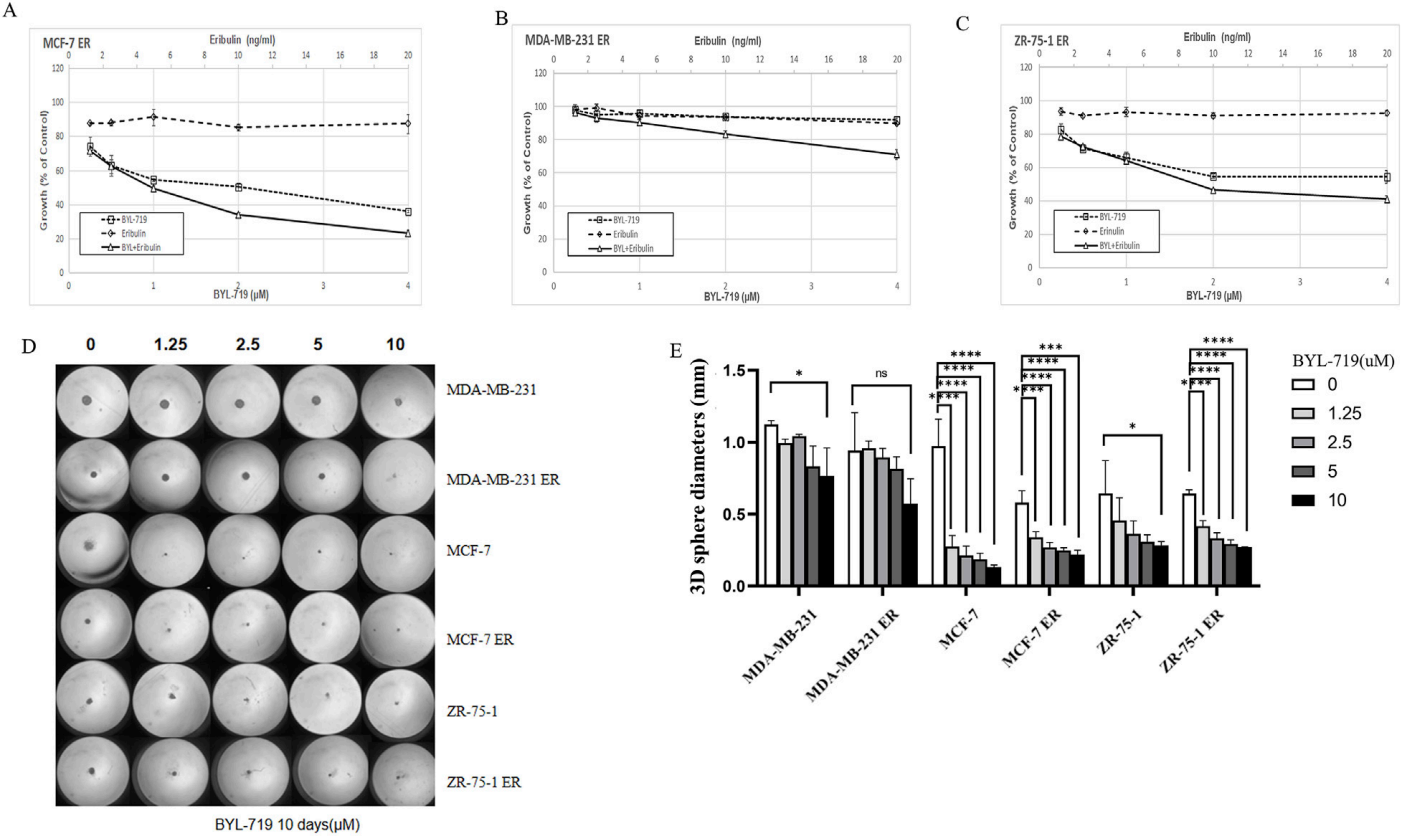
**(A–C)** BYL-719 could inhibit the cell viability of ER vells **(D, E)** Sphere-forming abilities of ER cells were all inhibited by BYL-719. Ns for non-significant, * for *P* < 0.05, ** for *P* < 0.01, *** for *P* < 0.001 and **** for *P* < 0.0001. Errors bars represent standard errors.

We analyzed whether the addition of BYL-719 to the culture increased the sensitivity of the cells to eribulin. MTT assays were performed to determine the effects of the combination of the two drugs. All the cell lines were proved to be resistant to eribulin via the MTT assays. Even extremely high concentration of eribulin had poor effects on the cell lines as shown in Figures 4A–C, while the combination groups of the BYL-719 and eribulin led to stronger cytotoxicity in ER cells compared to that with each drug alone, indicating that BYL-719 has the potential to increase the sensitivity of ER cells to eribulin.

Under 3D conditions, BYL-719 inhibited the growth and proliferation of ER-resistant cells in most cell lines by calculating the diameters of the spheres, except for the MDA-MB-231 ER cell group, which showed a high level of resistance (Figures 4D, E). These data indicated that there was no cross-resistance to BYL-719 in eribulin-resistant cells.

**FIGURE 5:**
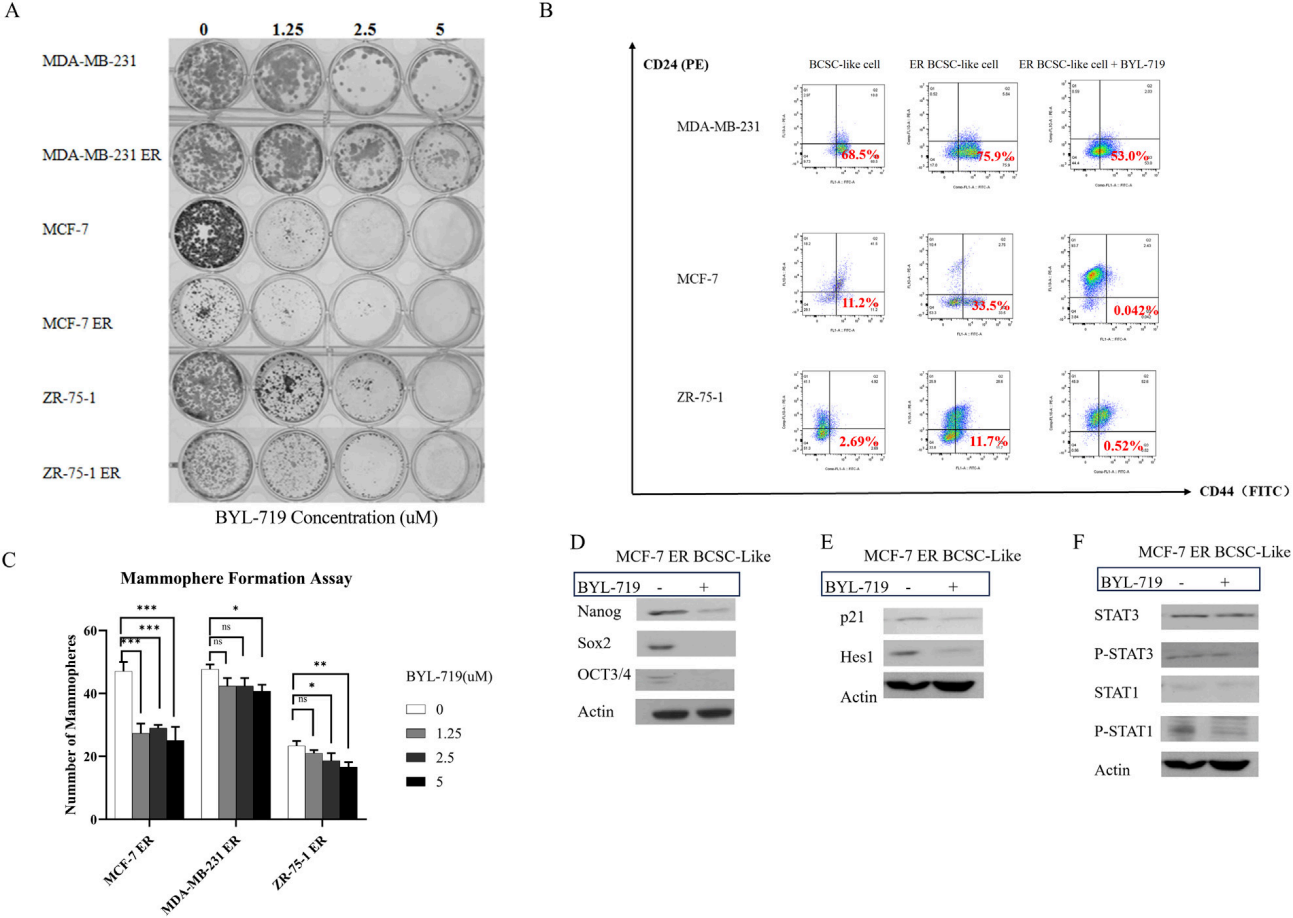
**(A)** BYL-719 efficiently inhibit colony-farming and stemness abilities of both native cell lines and ER cells. **(B)** BYL-719 reduced the subpopulation of CD44^+^CD24^−^ in ER treated groups. **(C)** BYL-719 effectively reduced the mammosphere formation efficiency in a dose dependent manner in Eribulin resistant cells. **(D)** EMT was inhibited by BYL-719 in MCF-7 ER treated group. **(E)** Notch signaling pathway and p21 protein were inhibited in MCF-7 ER treated group. **(F)** JAK/STAT signaling pathway was inhibited in MCF-7 ER treated group. **(B–F)** All the treated groups were treated with BYL-719 of the concentration of 1 μM for 24 h. Image **(E)** and Image **(F)** are from the same cells in a same experiment, with the same loading control. We seperate these images in order to explain the changes in different signaling pathways more clearly. Ns for non-significant, * for *P* < 0.05, ** for *P* < 0.01, *** for *P* < 0.001 and **** for *P* < 0.0001. Errors bars represent standard errors.

In the clonogenic assay, all six groups were inhibited by BYL-719, indicating that BCSC self-renewal and stemness were inhibited by BYL-719 (Figure 5A). Inhibition was more apparent in native breast cancer cell lines than in ER cells. These findings were attributed to the increased percentage of BCSC in the ER cells.

The mammosphere formation assay under 3D conditions (Figure 5C) showed that the number of mammospheres in ER cells significantly decreased after treatment with different doses of BYL-719 for 5 days. However, a significant reduction in the number of mammospheres was observed. Both the stemness of ER cells and their capacity to produce mammospheres decreased.

For further confirmation, we performed FACS sorting and western blotting to analyze the expression of surface and cytoplasmic BCSC markers in BCSC enriched ER cells under treatment with BYL-719. As shown in Figure 5B, we found that the subpopulation of cells with CD44^+^/CD24^−^surface markers in BCSC enriched in ER cells were reduced after treated by 1 μM BYL-719 for 24 h, indicating that even small dose of BYL-719 could have a direct effect on BCSCs. These differences were considered statistically significant, Nanog (*p* = 0.0152; 95% CI, 0.5036–2.662), SOX2 (*p* = 0.0116; 95% CI, 0.4989–2.200), and OCT3/4 (*p* = 0.0320; 95% CI, 0.05508–0.7305). These findings were confirmed using western blotting, which demonstrated that BYL-719 substantially reduced BCSC-related protein levels in BCSC-enriched MCF-7 ER cells (Figure 5D).

In MCF-7 ER cells, important signaling pathways, including Notch and STAT, which crosstalk with the PI3K/AKT/mTOR signaling pathway in the TME, showed significant changes (*p* < 0.05). Some changes were even more significant than those observed in native cells.

As shown in Figure 5E, by analyzing the Notch signaling pathway, p21 (*p* = 0.0361; 95% CI, 0.02892–0.3332) and Hes1 (*p* = 0.0372; 95% CI, 0.05508–0.7305) notably decreased in the MCF-7 ER BCSC-like group. These results confirmed our hypothesis that the Notch signaling pathway is active in BCSC and inhibited by BYL-719.

The results in Figure 5F showed P-STAT3 (*p* = 0.0466; 95% CI, 0.0090–0.4803) and P-STAT1 (*p* = 0.0459; 95% CI, 0.06989–3.057) were inhibited by BYL-719. However, we did not detect significant changes in STAT1 and STAT3. We still confirm that the STAT/JAK signaling pathway could also be inhibited by adding BYL-719 because phosphorylation in the Notch signaling pathway is closely associated with stem cells.

## 4 Discussion

### 4.1 The 3D-cell culture method is a novel and highly effective method to enrich BCSCs *in vitro*

While two-dimensional (2D) cell culture systems *in vitro* are widely used, they often fall short in accurately replicating physiological conditions relevant to clinical research. To address this limitation, in this experiment, we employed a three-dimensional (3D) mammosphere culture method, through which BCSC-like cells were harvested *in vitro*. These BCSC-like cells were harvested using a 3D cell culture method, whereby spheroids were derived from primary breast cancer cell lines and collected from differentiated cells (Habanjar et al., 2021). They are also referred to as “tumoroids” (Idrisova et al., 2022). The cell population is a subset of cells capable of dictating invasion, metastasis, heterogeneity, and therapeutic resistance in tumors (Jaggupilli and Elkord, 2012). CD44 and CD24 are important and widely recognized BCSC surface markers (Cataldo et al., 2024) which are found in many tumor types and are often used together or in combination with other putative markers to isolate stem cells from solid tumors (Jaggupilli and Elkord, 2012). This subpopulation of breast cancer cells (CD44^+^/CD24^−^) has stem/progenitor cell properties (Alvarez-Elizondo and Weihs, 2022). To quantify the ratio of CD44^+^/CD24^−^ subpopulation of cells, FACS was performed in this experiment. Moreover, BCSC-like cells are known to express higher levels of EMT, a phenomenon in which epithelial cells acquire mesenchymal properties, a process that has been observed in tumor progression and invasion (Savagner, 2010). Therefore, protein markers, including Nanog, OCT3/4, and SOX2, were detected, as they promote the emergence of CSCs with mesenchymal properties necessary for proliferation and self-renewal, which are required for secondary tumor formation (Vuoriluoto et al., 2011).

### 4.2 The stemness are inhibited by BYL-719 in both cell lines

Treatment with BYL-719 significantly inhibited the proliferation of BCSC-enriched mammosphere cultures 96 h after a single treatment (Figure 1A) and the IC50 values for BCSC-enriched mammosphere cultures increased by approximately two-fold for both cell lines. This indicates that stemness is indeed inhibited by BYL-719.

Furthermore, by performing an *in vitro* clonogenic assay, we detected cell survival. This is significant because clonogenic ability indicates the stemness of cancer cells, which is crucial in tumors where the capacity for unlimited proliferation can lead to tumor recurrences. As demonstrated in Figure 1B, both breast cancer cell lines exhibited an evident decrease in the number of colonies. Additionally, apoptosis was detected in both the control and treatment groups (Figure 1C). These findings suggest that BYL-719 is highly effective, as it promoted apoptosis in BCSC-like cells. This implies that BYL-719 could active and promote the cell death signaling pathways, which include autophagy, apoptosis, ferroptosis, and necroptosis through crosstalk among BCSC signaling pathways. Moreover, it inhibited anti-apoptotic mechanisms via survivin, Mcl-1, Bcl-2, IAPs, and DNA-repairing proteins (Kist and Vucic, 2021).

In the mammosphere formation assay, the volume of mammospheres decreased by the enhancing concentration of BYL-719, as shown in Figure 2A. Under the 3D condition, the diameters of spheres were inhibited, as shown in Figure 2B. Additionally, the stem cell surface marker CD44 and CD24 were detected, and the proportion of the subpopulation (CD44^+^/CD24^−^) were then calculated by FACS analysis (Figure 2C). In both cell lines, the proportion of CD44^+^/CD24^−^ cells significantly decreased after treatment with 1 μM BYL-719 for 24 h (Figure 2D). By comparing the western blot of MCF-7 BCSC-like and MCF-7 BCSC-like cells in 1 μM BYL-719 for 48 h, the decrease of Nanog, SOX2 and OCT3/4 were statistically significant (Figure 2E).

### 4.3 BYL-719 inhibited the stemness by acting on various signaling pathways which play essential roles in the TME of BCSCs

The PI3K/mTOR signaling pathway is essential for cell survival and proliferation (Glaviano et al., 2023). In fact, some malignancies, such as non-small-cell lung, breast, prostate, and colorectal cancer, exhibit an abnormal activation of PI3K/AKT/mTOR signaling (Jiang et al., 2020; Yu et al., 2022). Although the PI3K/AKT/mTOR pathway has been extensively investigated in cancer, few studies have been conducted on CSCs (Yoon et al., 2024). Blocking the PI3K signaling pathway to stop tumor growth is not a new concept; many inhibitors, such as two rapalogues, everolimus, and temsirolimus, have been used for many years with good efficacy (Bai et al., 2018). In our study, we confirmed the inhibition of PI3K by decreasing p-P70S6K; p-4EBP1, p-AKT, and pARP (Figure 3A).

Moreover, other major signaling pathways are also involved in CSC self-renewal and differentiation, including the Notch, MAPK/ERK, JAK-STAT, Wnt/β-catenin, and Hedgehog (Hh) signaling pathways (Bhal and Kundu, 2023). Additionally, other important signaling pathways in CSCs include TNF-α/NF-κβ, transforming growth factor-β (TGF-β), and receptor tyrosine kinase (RTK) signaling pathways (Chia et al., 2024; Borlongan and Wang, 2023). These signaling pathways are related to self-renewal and differentiation (Xu et al., 2018). Western blotting was performed to detect changes in important proteins in the MAPK/ERK, Notch, and JAK-STAT signaling pathways, which were confirmed to be important in BCSCs (Figures 3B–E). The mitogen-activated protein kinase/extracellular signal-regulated kinase (MAPK/ERK) pathway is associated with cell proliferation, differentiation, migration, aging, and apoptosis (Sun et al., 2015). Similarly, the Notch signaling pathway also plays an important role in cell development and differentiation (Shi et al., 2024). Furthermore, the JAK-STAT signaling pathway is also activated in BCSCs, and persistent activation of STAT3 can stimulate cell survival and maintain stem cell properties in BCSCs (Zhou et al., 2007). Interestingly, the PI3K/mTOR pathway regulates STAT3 expression and promotes the survival and proliferation of BCSCs (Zhou et al., 2007). In our study, important downstream proteins were detected, and we confirmed the significant inhibition of all these signaling pathways.

### 4.4 BYL-719 could help overcome the drug resistance of breast cancer cells by acting on BCSCs

In the final phase of our experiment, the function and influence of BYL-719 to the resistance of tumor cells were the focus. To explore this, several drug-resistant cell lines were also cultured. Specifically, three eribulin-resistant cell lines, including–MCF-7 ER, MDA-MB-231 ER, and ZR-75-1 ER–were used in this study. These cell lines were cultured, collected, and confirmed to be highly resistant to eribulin, a non-taxane microtubule dynamics inhibitor with tubulin-based anti-mitotic activity and chemotherapeutic effects (Perry, 2011).

As is well-documented, CSCs can easily adapt to environmental changes and are inherently more resistant to conventional therapies compared to other cells in the tumor (Najafi et al., 2019). This drug resistance in CSCs could be secondary to radiotherapy or chemotherapy or may even be induced after isolation from chemotherapy (Prieto-Vila et al., 2017). The combination of intrinsic and extrinsic factors significantly contributes to the CSC-mediated resistance to treatment (Najafi et al., 2019). Intrinsic factors include EMT, oxidative regulators, stem cells, and signaling effects, whereas extrinsic factors include cellular plasticity and some signaling factors (Najafi et al., 2019). To overcome clinical resistance to treatment, scientists have focused on novel insights into CSCs. While standard therapies act on rapidly dividing cells and are generally effective in reducing the size of the primary tumor, complete eradication remains challenging due to the presence of CSCs. Despite their high proliferative capacity, CSCs, such as BCSCs, spend most of their time in a resting state (cell cycle phase G0), which allows them to protect themselves from chemotherapy and radiation (Zou et al., 2011). Moreover, the relative resistance of BCSCs to radiation and cytotoxic agents may be due to a more efficient DNA damage response mechanism, which can result in less cell death than that in other breast cell types (Saha and Lukong, 2022; Chang et al., 2015). Furthermore, BCSCs are more resistant to radiotherapy and chemotherapy due to their abundance in hypoxic regions (Chang et al., 2015). The fact that BCSCs are resistant to standard therapies highlights the need for novel therapies targeting BCSC populations. These distinct characteristics, markers, and resistance mechanisms suggest that targeting BCSCs is an essential breakthrough for developing more effective therapies for patients with breast cancer, either alone or in combination with currently used therapies.

Currently, BCSCs are suggested to be novel and essential targets for clinical BCSC therapy to overcome drug resistance and relapse. Emerging findings regarding surface markers and signaling networks support the development of therapeutic approaches using BCSCs (Conde et al., 2022) as a target. In future, we will do more experiments at the gene level and *in vivo* animal experiments will be performed. Additionally, we intend to explore the combination of BYL-719 with other chemotherapeutic agents. Our hope is to identify the related genes of BCSCs in future.

## 5 Conclusion

Our research demonstrated that BYL-719 has a significant effect on BCSCs, and that the combination of BYL-719 with eribulin further overcomes drug resistance. We are optimistic that these findings will contribute to the development of therapies that can directly target BCSCs, thereby reducing metastasis and relapse in breast cancer. We believe that drug-resistant patients will benefit from the combination of BYL-719 with other chemotherapeutic agents in future studies.

## Data Availability

The raw data supporting the conclusions of this article will be made available by the authors, without undue reservation.
